# The treatment responses among different inhalation therapies for GOLD group E patients with chronic obstructive pulmonary disease

**DOI:** 10.7189/jogh.15.04055

**Published:** 2025-02-21

**Authors:** Qing Song, Ling Lin, Tao Li, Ping Zhang, Yuqin Zeng, Dingding Deng, Rong Yi, Dan Liu, Yan Chen, Shan Cai, Ping Chen, Cong Liu

**Affiliations:** 1 Department of Pulmonary and Critical Care Medicine, the Second Xiangya Hospital, Central South University, Changsha, Hunan, China; 2 Research Unit of Respiratory Disease, Central South University, Changsha, Hunan, China; 3 Clinical Medical Research Center for Pulmonary and Critical Care Medicine in Hunan Province, Changsha, Hunan, China; 4 Diagnosis and Treatment Center of Respiratory Disease in Hunan Province, Changsha, Hunan, China; 5 Department of Respiratory and Critical Care Medicine, First Affiliated People's Hospital of Shaoyang University, Shaoyang, Hunan, China; 6 Department of Pulmonary and Critical Care Medicine, Zhuzhou Central Hospital, Zhuzhou, Hunan, China; 7 Department of Respiratory and Critical Care Medicine, Changsha Eighth Hospital, Changsha, Hunan, China

## Abstract

**Background:**

The Global Initiative for Chronic Obstructive Lung Disease (GOLD) 2023 revised the combined chronic obstructive pulmonary disease (COPD) assessment, merging groups C and D into group E, and revised the initial inhalation therapy recommendation. We aimed to evaluate the treatment responses among different inhalation therapies in GOLD group E patients stratified by the COPD assessment test (CAT) scores and forced expiratory volume in one-second percentage of predicted (FEV1%pred).

**Methods:**

In this retrospective cohort study, we included patients with COPD registered in the Real World Research of Diagnosis and Treatment of COPD (RealDTC) study between January 2017 and June 2023. According to the GOLD 2023 report, we enrolled patients assigned to GOLD group E based on exacerbations in the past year (≥2 exacerbations or ≥1 hospitalisation) in this study. We classified them into the FEV1%pred <50% and FEV1%pred ≥50% groups, or CAT<10 and CAT≥10 groups. Subsequently, we divided all groups into four subgroups: long-acting muscarinic antagonist (LAMA), long-acting β2-agonist (LABA) + inhaled corticosteroid (ICS), LABA + LAMA, and LABA + LAMA + ICS. All patients finished one year of follow-up, during which we collected data on exacerbations, frequent exacerbations, hospitalisations, and all-cause mortality. We defined frequent exacerbations as ≥2 exacerbations per year.

**Results:**

We enrolled a total of 3173 patients in this study. During one year of follow-up, there were no significant differences in exacerbations, frequent exacerbations, hospitalisations, and all-cause mortality among LAMA, LABA + LAMA, LABA + ICS, and LABA + LAMA + ICS in the FEV1%pred ≥50% and CAT<10 groups. However, the patients treated with LABA + LAMA or LABA + LAMA + ICS had a lower incidence of exacerbations and frequent exacerbations compared with the patients treated with LAMA or LABA + ICS in the FEV1%pred <50% and CAT≥10 groups (*P* < 0.05).

**Conclusions:**

Patients with COPD in GOLD group E should be further stratified to determine the appropriate initial inhalation therapy. This approach may provide more precise treatment for GOLD group E patients.

Chronic obstructive pulmonary disease (COPD) is a complex and heterogeneous chronic respiratory disease, characterised by a high morbidity and mortality that make it the third leading cause of death globally [1,2]. As precise, up-to-date treatment for is vital to improve outcomes among affected patients, the Global Initiative for Chronic Obstructive Lung Disease (GOLD) programme provides annual updates on recommended strategies for managing COPD. Until GOLD 2011, the combined assessment tool classified patients into the A, B, C, and D groups based on the COPD assessment test (CAT) or the modified Medical Research Council (mMRC), the risk of exacerbation history, and forced expiratory volume in the one-second percentage of predicted (FEV1%pred) [3]. However, the GOLD 2023 report revised the combined COPD assessment tool, whereby the patients who suffered ≥2 exacerbations or ≥1 leading to hospitalisation were assigned to group E. It also introduced a change whereby patients in GOLD group E should be offered a long-acting β2-agonist (LABA) + long-acting muscarinic antagonist (LAMA), or a LABA + LAMA + inhaled corticosteroid (ICS) as the initial inhalation therapy [4]. While this new grouping could simplify clinical assessment and management and optimise treatment recommendations for COPD patients, exacerbation history alone is not sufficient to summarise the complexity of management and treatment in GOLD group E patients due to the heterogeneity of COPD. The CAT score covers eight dimensions and is better than the mMRC score in assessing the symptoms of COPD patients and is closely related to their progression [5–7]. In addition, FEV1%pred is an important indicator for assessing the severity of COPD and is also closely related to the prognosis of COPD patients [8–10].

Therefore, we aimed to evaluate the treatment responses among different inhalation therapies in GOLD group E patients stratified by the CAT score and FEV1%pred.

## METHODS

### Study participants

This was a multicentre retrospective cohort study performed on a sample of patients registered in the Real World Research of Diagnosis and Treatment of COPD (RealDTC) study between January 2017 and June 2023 [11]. The patients had been diagnosed with COPD according to the GOLD 2017 report: the ratio of forced expiratory volume in one second to forced vital capacity (FEV1/FVC) was <0.70 after inhaling a bronchodilator [12]. We excluded patients with active tuberculosis, bronchiectasis, lung cancer, pneumonia, pulmonary fibrosis, and severe heart, liver, or kidney disease.

### Data collection

We collected the baseline data, including age, sex, body mass index (BMI), FEV1%pred, FEV1/FVC, GOLD grade, CAT score, mMRC score, exacerbations in the past year, inhalation therapy, and comorbidities (including chronic heart disease, hypertension, and diabetes) when patients first visited hospitals. Patients in the RealDTC were followed up every six months from their first hospital visit and the information on the number of exacerbations and death was recorded. All patients have finished one year of follow-up to collect data on exacerbations, frequent exacerbations, hospitalisations, and all-cause mortality.

### Study procedures

According to the GOLD 2023 report, we enrolled patients who were assigned to GOLD group E based on exacerbations in the past year (≥2 exacerbations or ≥1 hospitalisation) [4]. According to the baseline data when the patients first visited hospitals, we classified them into four groups: FEV1%pred <50% and FEV1%pred ≥50% groups, or CAT<10 and CAT≥10 groups. Subsequently, we divided each group into the LAMA, LABA + ICS, LABA + LAMA, and LABA + LAMA + ICS subgroups based on the initial inhalation therapies the patients received.

### Definition of variables

An exacerbation is a COPD progression that requires antibiotics, oral corticosteroids, or hospitalisation [13]. We defined frequent exacerbations as ≥2 exacerbations per year [14]. We classified a current-smoker as someone who smoked ≥10 packs/y and a former-smoker as someone who smoked ≥10 packs/y but had not smoked for more than six months [15]. GOLD grades were based on the post-bronchodilator FEV1%pred as follows: GOLD grade one (≥80%), GOLD grade two (50–79%), GOLD grade three (30–49%), GOLD grade four (<30%) [4]. We defined the prescription outcomes (including adjusted treatment) as a change in the inhalation therapy drugs or cessation of inhalation therapy drugs for more than three months during one year of follow-up [16,17].

### Statistical analysis

We used SPSS, version 26.0 (IBM Corp., Armonk, New York, USA) and Free Statistics software, version 1.7.1 (Beijing, China) for statistical analysis. We expressed continuous variables with a normal distribution and homogeneity of variance as means (x̄) and standard deviations (SDs) and analysed them using variance analysis. Variables that did not meet these assumptions were summarised as medians (MDs) and interquartile ranges (IQRs) and were analysed with non-parametric tests. We used the chi-squared (χ^2^) test or Fisher exact test to analyse categorical variables. We calculated the adjusted odds ratio (aOR) and its 95% confidence intervals (95% CI) using multiple logistic regression. Variables in the logistic regression model include therapy, age, sex, biofuel exposure, smoke history, BMI, FEV1%pred, FEV1/FVC, CAT, mMRC, exacerbation in the past year, prescription outcomes, and comorbidities. There was no collinearity among the variables in the logistic regression model (Table S1 in the **Online Supplementary Document**). We considered *P*-values <0.05 as statistically significant.

## RESULTS

### Baseline characteristics of COPD patients

We included 3173 patients with COPD (Figure S1 in the **Online Supplementary Document**). The mean age was 66.7 years (SD = 8.9), with most of the patients being male (n = 2701, 85.1%) (**Table 1**). A total of 720 (22.7%) patients received LAMA, 362 (11.4%) LABA + LAMA, 423 (13.3%) LABA + ICS, and 1668 (52.6%) received LABA + LAMA + ICS.

**Table 1:** Baseline characteristics of COPD patients*

	Values
**Total number of participants**	3173
**Age (years), x̄ (SD)**	66.7 (8.9)
**Sex**	
Male	2701 (85.1)
Female	472 (14.9)
**BMI (kg/m^2^), x̄ (SD)**	22.4 (3.6)
**Smoke history**	
Never smoker	754 (23.8)
Former smoker	1184 (37.3)
Current smoker	1235(38.9)
**Smoking (pack/y), MD (IQR)**	35 (10–50)
**Biofuel exposure**	
Yes	1312 (41.3)
No	1861 (58.7)
**FEV1%pred, x̄ (SD)**	50.7 (19.6)
**FEV1/FVC, x̄ (SD)**	46.3 (12.1)
**GOLD grades**	
1	260 (8.2)
2	1226 (38.6)
3	1238 (39.0)
4	449 (14.2)
**CAT, x̄ (SD)**	16.4 (6.7)
**CAT**	
<10	509 (16.0)
10–19	1705 (53.7)
20–29	847 (26.7)
≥30	112 (3.6)
**mMRC, MD (IQR)**	2 (1–3)
**mMRC**	
0–1	820 (25.8)
2–4	2353 (74.2)
**Therapy**	
LAMA	720 (22.7)
LABA + LAMA	362 (11.4)
LABA + ICS	423 (13.3)
LABA + LAMA + ICS	1668 (52.6)
**Exacerbations in the past year, MD (IQR)**	1 (1–4)
**Exacerbations in the past year**	
1	1011 (31.9)
≥2	2162 (68.1)
**Hospitalisations in the past year, MD (IQR)**	1 (0–2)
**Hospitalisations in the past year**	
No	937 (29.5)
Yes	2236 (70.5)
**Comorbidities**	
Chronic heart disease	89 (2.8)
Hypertension	102 (3.2)
Diabetes	41 (1.3)

BMI – body mass index, CAT – COPD assessment test, COPD – chronic obstructive pulmonary disease, FEV1%pred – forced expiratory volume in the first-second percentage of predicted, FVC – forced vital capacity, GOLD – global initiative for chronic obstructive lung disease, ICS – inhaled corticosteroid, IQR – interquartile range, LABA – long-acting β2-agonist, LAMA – long-acting muscarinic antagonist, MD – median, mMRC – modified medical research council, SD – standard deviation, x̄ – mean

*Presented as n (%) unless specified otherwise.

During one year of follow-up, 38.8% of patients experienced exacerbations, 19.8% had frequent exacerbations, and 23.3% were hospitalised (**Table 2**). The all-cause mortality rate was 2.2%.

**Table 2:** Treatment responses in COPD patients during one year of follow-up*

	Values
**Total number of participants**	3173
**Exacerbations, MD (IQR)**	0 (0–1)
**Exacerbations**	
Yes	1204 (38.8)
No	1898 (61.2)
**Frequent exacerbations**	
Yes	615 (19.8)
No	2487 (80.2)
**Hospitalisations, MD (IQR)**	0 (0–0)
**Hospitalisations**	
Yes	724 (23.3)
No	2378 (76.7)
**All-cause mortality**	
Yes	71 (2.2)
No	3102 (97.8)
**Prescription outcomes**	
Adjust treatment	722 (22.8)
Continuous using	2451 (77.2)

COPD – chronic obstructive pulmonary disease, IQR – interquartile range, MD – median

*Presented as n (%) unless specified otherwise.

### Treatment responses among different inhalation therapies in FEV1%pred ≥50% group

There were 1486 patients in the FEV1%pred ≥50% group, assigned to the LAMA (n = 453, 30.5%), LABA + LAMA (n = 178, 12.0%), LABA + ICS (n = 285, 19.2%), and LABA + LAMA + ICS (n = 570, 38.3%) subgroups (**Table 3**). After adjusting for the confounding factors (*i.e*. age, sex, BMI, smoke history, biofuel exposure, FEV1%pred, FEV1/FVC, CAT, mMRC, exacerbations in the past year, prescription outcomes, and comorbidities), there were no significant differences in exacerbations, frequent exacerbations, hospitalisations, and all-cause mortality among any of the three groups (Tables S2 and S3 in the **Online Supplementary Document**).

**Table 3:** Treatment responses among different inhalation therapies in FEV1%pred ≥50% group*

	Total	LAMA	LABA + LAMA	LABA + ICS	LABA + LAMA + ICS	*P*-value
**Total number of participants**	1486	453 (30.5)	178 (12.0)	285 (19.2)	570 (38.3)	
**Exacerbations, MD (IQR)**	0 (0–1)	0 (0–1)	0 (0–1)	0 (0–1)	0 (0–1)	0.304
**Exacerbations**						0.684
Yes	562 (38.4)	183 (41.1)	60 (34.7)	100 (35.8)	219 (38.7)	
No	901 (61.6)	262 (58.9)	113 (65.3)	179 (64.2)	347 (61.3)	
**Frequent exacerbations**						0.258
Yes	272 (18.6)	96 (21.6)	30 (17.3)	51 (18.3)	95 (16.8)	
No	1191 (81.4)	349 (78.4)	143 (82.7)	228 (81.7)	471 (83.2)	
**Hospitalisations, MD (IQR)**	0 (0–0)	0 (0–0)	0 (0–0)	0 (0–0)	0 (0–1)	0.058
**Hospitalisations**						0.080
Yes	330 (22.6)	98 (22)	39 (22.5)	49 (17.6)	144 (25.4)	
No	1133 (77.4)	347 (78)	134 (77.5)	230 (82.4)	422 (74.6)	
**All-cause mortality**						0.093
Yes	23 (1.5)	8 (1.8)	5 (2.8)	6 (2.1)	4 (0.7)	
No	1463 (98.5)	445 (98.2)	173 (97.2)	279 (97.9)	566 (99.3)	
**Prescription outcomes**						0.003
Adjust treatment	380 (25.6)	143 (31.6)	36 (20.2)	62 (21.8)	139 (24.4)	
Continuous using	1106 (74.4)	310 (68.4)	142 (79.8)	223 (78.2)	431 (75.6)	

FEV1%pred – forced expiratory volume in the first-second percentage of predicted, ICS – inhaled corticosteroid, IQR – interquartile range, LABA – long-acting β2-agonist, LAMA – long-acting muscarinic antagonist, MD – median

*Presented as n (%) unless specified otherwise.

### Treatment responses among different inhalation therapies in FEV1%pred <50% group

There were 1687 patients in the FEV1%pred <50% group, assigned to the LAMA (n = 267, 15.8%), LABA + LAMA (n = 184, 10.9%), LABA + ICS (n = 138, 8.2%), and LABA + LAMA + ICS (n = 1098, 65.1%) subgroups (**Table 4**). After adjusting for the confounding factors, patients treated with LAMA or LABA + ICS had a higher incidence of exacerbations (aOR = 1.74; 95% CI = 1.17–2.61, *P* = 0.007 and aOR = 1.48; 95% CI = 1.01–2.35, *P* = 0.015, respectively) and frequent exacerbations (aOR = 2.55; 95% CI = 1.53–4.26, *P* < 0.001 and aOR = 2.27; 95% CI = 1.27–4.06, *P* = 0.006, respectively) compared to patients treated with LABA + LAMA (Table S4 in the **Online Supplementary Document**).

**Table 4:** Treatment responses among different inhalation therapies in FEV1%pred <50% group*

	Total	LAMA	LABA + LAMA	LABA + ICS	LABA + LAMA + ICS	*P*-value
**Total number of participants**	1687	267 (15.8)	184 (10.9)	138 (8.2)	1098 (65.1)	
**Exacerbations, MD (IQR)**	0 (0–1)	0 (0–2)	0 (0–1)	0 (0–2)	0 (0–1)	<0.001
**Exacerbations**						<0.001
Yes	642 (39.2)	128 (49.2)	64 (35.8)	61 (44.9)	389 (36.6)	
No	997 (60.8)	132 (50.8)	115 (64.2)	75 (55.1)	675 (63.4)	
**Frequent exacerbations**						<0.001
Yes	1296 (79.1)	184 (70.8)	154 (86)	100 (73.5)	858 (80.6)	
No	343 (20.9)	76 (29.2)	25 (14)	36 (26.5)	206 (19.4)	
**Hospitalisations, MD (IQR)**	0 (0–0)	0 (0–0)	0 (0–0)	0 (0–0)	0 (0–0)	0.718
**Hospitalisations**						0.758
Yes	1245 (76.0)	200 (76.9)	141 (78.8)	103 (75.7)	801 (75.3)	
No	394 (24.0)	60 (23.1)	38 (21.2)	33 (24.3)	263 (24.7)	
**All-cause mortality**						0.838
Yes	48 (2.8)	7 (2.6)	5 (2.7)	2 (1.4)	34 (3.1)	
No	1639 (97.2)	260 (97.4)	179 (97.3)	136 (98.6)	1064 (96.9)	
**Prescription outcomes**						0.087
Adjust treatment	342 (20.3)	64 (24)	26 (14.1)	28 (20.3)	224 (20.4)	
Continuous using	1345 (79.7)	203 (76)	158 (85.9)	110 (79.7)	874 (79.6)	

FEV1%pred – forced expiratory volume in the first-second percentage of predicted, ICS – inhaled corticosteroid, IQR – interquartile range, LABA – long-acting β2-agonist, LAMA – long-acting muscarinic antagonist, MD – median

*Presented as n (%) unless specified otherwise.

Patients treated with LAMA or LABA + ICS had a higher incidence of exacerbations (aOR = 1.74; 95% CI = 1.31–2.31, *P* < 0.001 and aOR = 1.47; 95% CI = 1.01–2.13, *P* = 0.043, respectively) and frequent exacerbations (aOR = 1.81; 95% CI = 1.32–2.50, *P* < 0.001 and aOR = 1.62; 95% CI = 1.06–2.47, *P* = 0.027, respectively) compared to patients treated with LABA + LAMA + ICS (Table S5 in the **Online Supplementary Document**).

### Treatment responses among different inhalation therapies in CAT<10 group

There were 509 patients in the CAT<10 group, assigned to the LAMA (n = 145, 28.5%), LABA + LAMA (n = 70, 13.8%), LABA + ICS (n = 118,  23.2%), and LABA + LAMA + ICS (n = 176, 34.5%) subgroups (**Table 5**). After adjusting for the confounding factors (*i.e.* age, sex, BMI, smoke history, biofuel exposure, FEV1%pred, FEV1/FVC, CAT, mMRC, exacerbations in the past year, prescription outcomes, and comorbidities), there were no significant differences in exacerbations, frequent exacerbations, hospitalisations, and all-cause mortality among LAMA, LABA + LAMA, LABA + ICS, and LABA + LAMA + ICS (Tables S6 and S7 in the **Online Supplementary Document**).

**Table 5:** Treatment responses among different inhalation therapies in CAT<10 group*

	Total	LAMA	LABA + LAMA	LABA + ICS	LABA + LAMA + ICS	*P*-value
**Total number of participants**	509	145 (28.5)	70 (13.8)	118 (23.2)	176 (34.5)	
**Exacerbations, MD (IQR)**	0 (0–1)	0 (0–1)	0 (0–1)	0 (0–1)	0 (0–1)	0.694
**Exacerbations**						0.486
Yes	187 (37.0)	54 (37.8)	28 (40.0)	36 (31.0)	69 (39.2)	
No	318 (63.0)	89 (62.2)	42 (60.0)	80 (69.0)	107 (60.8)	
**Frequent exacerbations**						0.987
Yes	81 (16.0)	23 (16.1)	12 (17.1)	19 (16.4)	27 (15.3)	
No	424 (84.0)	120 (83.9)	58 (82.9)	97 (83.6)	149 (84.7)	
**Hospitalisations, MD (IQR)**	0 (0–0)	0 (0–0)	0 (0–0)	0 (0–0)	0 (0–1)	0.286
**Hospitalisations**						0.310
Yes	113 (22.4)	32 (22.4)	17 (24.3)	19 (16.4)	45 (25.6)	
No	392 (77.6)	111 (77.6)	53 (75.7)	97 (83.6)	131 (74.4)	
**All-cause mortality**						0.267
Yes	4 (0.8)	2 (1.4)	0 (0.0)	2 (1.7)	0 (0.0)	
No	505 (99.2)	143 (98.6)	70 (100.0)	116 (98.3)	176 (100.0)	
**Prescription outcomes**						0.042
Adjust treatment	131 (25.7)	49 (33.8)	12 (17.1)	28 (23.7)	42 (23.9)	
Continuous using	378 (74.3)	96 (66.2)	58 (82.9)	90 (76.3)	134 (76.1)	

CAT – COPD assessment test, COPD – chronic obstructive pulmonary disease, ICS – inhaled corticosteroid, IQR – interquartile range, LABA – long-acting β2-agonist, LAMA – long-acting muscarinic antagonist, MD – median

*Presented as n (%) unless specified otherwise.

### Treatment responses among different inhalation therapies in CAT≥10 group

There were 2664 patients in the CAT≥10 group, assigned to the LAMA (n = 575, 21.6%), LABA + LAMA (n = 292, 11.0%), LABA + ICS (n = 305, 11.4%), and LABA + LAMA + ICS (n = 1492, 56.0%) subgroups (**Table 6**). After adjusting for the confounding factors, the patients treated with LAMA or LABA + ICS had a higher incidence of exacerbations (aOR = 1.69; 95% CI = 1.24–2.29, *P* = 0.001 and aOR = 1.48; 95% CI = 1.05–2.09, *P* = 0.024, respectively) and frequent exacerbations (aOR = 2.01; 95% CI = 1.37–2.95, *P* < 0.001 and aOR = 1.73; 95% CI = 1.12–2.66, *P* = 0.013, respectively) compared to the patients treated with LABA + LAMA (Table S8 in the **Online Supplementary Document**).

**Table 6:** Treatment responses among different inhalation therapies in CAT≥10 group*

	Total	LAMA	LABA + LAMA	LABA + ICS	LABA + LAMA + ICS	*P*-value
**Total number of participants**	2664	575 (21.6)	292 (11.0)	305 (11.4)	1492 (56.0)	
**Exacerbations, MD (IQR)**	0 (0–1)	0 (0–2)	0 (0–1)	0 (0–2)	0 (0–1)	<0.001
**Exacerbations**						<0.001
Yes	1017 (39.2)	257 (45.7)	96 (34)	125 (41.8)	539 (37.1)	
No	1580 (60.8)	305 (54.3)	186 (66)	174 (58.2)	915 (62.9)	
**Frequent exacerbations**						<0.001
Yes	534 (20.6)	149 (26.5)	43 (15.2)	68 (22.7)	274 (18.8)	
No	2063 (79.4)	413 (73.5)	239 (84.8)	231 (77.3)	1180 (81.2)	
**Hospitalisations, MD (IQR)**	0 (0–0)	0 (0–0)	0 (0–0)	0 (0–0)	0 (0–0)	0.256
**Hospitalisations**						0.296
Yes	611 (23.5)	126 (22.4)	60 (21.3)	63 (21.1)	362 (24.9)	
No	1986 (76.5)	436 (77.6)	222 (78.7)	236 (78.9)	1092 (75.1)	
**All-cause mortality**						0.678
Yes	67 (2.5)	13 (2.3)	10 (3.4)	6 (2)	38 (2.5)	
No	2597 (97.5)	562 (97.7)	282 (96.6)	299 (98)	1454 (97.5)	
**Prescription outcomes**						0.002
Adjust treatment	591 (22.2)	158 (27.5)	50 (17.1)	62 (20.3)	321 (21.5)	
Continuous using	2073 (77.8)	417 (72.5)	242 (82.9)	243 (79.7)	1171 (78.5)	

CAT – COPD assessment test, COPD – chronic obstructive pulmonary disease, ICS – inhaled corticosteroid, IQR – interquartile range, LABA – long-acting β2-agonist, LAMA – long-acting muscarinic antagonist, MD – median

*Presented as n (%) unless specified otherwise.

Patients treated with LAMA or LABA + ICS had a higher incidence of exacerbations (aOR = 1.51; 95% CI = 1.23–1.86, *P* < 0.001 and aOR = 1.36; 95% CI = 1.04–1.77, *P* = 0.023, respectively) and frequent exacerbations (aOR = 1.70; 95% CI = 1.33–2.17, *P* < 0.001 and aOR = 1.46; 95% CI = 1.07–2.00, *P* = 0.018, respectively) compared to patients treated with LABA + LAMA + ICS (Table S9 in the **Online Supplementary Document**).

## DISCUSSION

The GOLD report is the most widely accepted guideline for managing and treating COPD, while the combined COPD assessment used within it is crucial for evaluating patients and recommending the most appropriate initial inhalation therapy. Notably, the GOLD 2023 report introduced a significant revision to the COPD combined assessment, merging groups C and D into a new group E to emphasise the clinical importance of exacerbations. According to the GOLD 2023 report, LABA + LAMA or LABA + LAMA + ICS is the preferred initial therapy for patients in group E [4,18]. This new classification simplifies clinical assessment and optimises treatment recommendations. However, COPD is a heterogeneous disease, and relying solely on exacerbation history may not fully capture the complexity of managing and treating group E patients. While this approach makes treatment selection straightforward for clinicians, its broad classification may limit the precision of therapy. Furthermore, previous studies have shown that nearly 50% of COPD patients in real-world settings fall into group E [15,19,20]. Therefore, analysing treatment responses among different inhalation therapies after stratifying group E patients is of significant clinical importance.

Although the GOLD report recommends initial inhalation therapy for group E COPD patients, clinicians do not always follow these guidelines in real-world practice. As a result, LAMA, LABA + LAMA, LABA + ICS, and LABA + LAMA + ICS are all potential initial therapies for group E patients [19]. To our knowledge, we are the first to analyse treatment responses among different inhalation therapies in GOLD group E after stratifying patients by CAT score and FEV1%pred. Our findings show that patients with FEV1%pred <50% and CAT≥10 treated with LABA + LAMA or LABA + LAMA + ICS had a lower incidence of exacerbations and frequent exacerbations compared to those treated with LAMA or LABA + ICS. These findings align with the GOLD report’s recommendations, supporting LABA + LAMA or LABA + LAMA + ICS as the preferred treatment for group E patients. Given that these patients have severe or very severe COPD and more symptoms, stronger inhalation therapy is necessary to improve outcomes, and LABA + LAMA is superior to LAMA or LABA + ICS [21]. Previous research also supports our findings, showing that patients treated with LABA + LAMA or LABA + LAMA + ICS had a lower incidence of exacerbation and frequent exacerbation compared with the patients treated with LAMA or LABA + ICS in group D [17]. Additionally, LABA + LAMA + ICS, rather than LABA + LAMA, should be recommended for patients with a blood eosinophil count ≥300 cells/μL or a history of asthma, as these patients derive greater benefit from ICS [4]. Suissa et al. [22] found that GOLD group E patients with a blood eosinophil count ≥300 cells/μL who were treated with LABA + LAMA + ICS had fewer moderate or severe exacerbations compared to those treated with LABA + LAMA. Moreover, ICS is the preferred anti-inflammatory treatment for asthma, and the GOLD report strongly recommends its use in COPD patients with a history of asthma [4].

However, in patients with FEV1%pred ≥50% and CAT<10, we found no significant differences in exacerbations, frequent exacerbations, and hospitalisations among LAMA, LABA + LAMA, LABA + ICS, and LABA + LAMA + ICS. This suggests that mono-LAMA may be a more appropriate initial therapy for these patients. A previous study also found no significant differences in exacerbations, hospitalisations, and mortality among these treatments in COPD patients with fewer symptoms (CAT<10 or mMRC<2) [23], supporting our findings. This may be because these patients have mild to moderate COPD and fewer symptoms, and mono-LAMA is sufficient to control their condition. Our findings highlight the importance of stratifying GOLD group E patients by CAT score and FEV1%pred when selecting initial inhalation therapy. A stratified approach can provide more precise treatment, reduce the risk of exacerbation, lower medical costs, and prevent overtreatment. Finally, the all-cause mortality rate in our study was 2.2%, consistent with previous research [19,24,25]. We found no significant differences in all-cause mortality among different inhalation therapies in GOLD group E patients stratified by CAT score and FEV1%pred, possibly due to the relatively short follow-up period in our study.

This study has several limitations. First, as a retrospective cohort and real-world study, it could not provide cause-and-effect explanations inherent to randomised controlled trials, which are needed to validate our findings in the future. Additionally, we did not analyse treatment responses among different inhalation therapies in group E after stratifying patients based on other treatable traits, such as eosinophil count, and comorbidities. Future research should consider these factors to provide a more comprehensive understanding of treatment outcomes.

## CONCLUSIONS

Patients with COPD in GOLD group E should be further stratified to identify the most appropriate initial inhalation therapy. A stratified approach could enable more precise treatment for GOLD group E patients, optimising patient outcomes and improving disease management.

## Additional material


Online Supplementary Document
