# Supplementary material for: The treatment responses among different inhalation therapies for GOLD group E patients with chronic obstructive pulmonary disease
Source: J Glob Health. 2025 Feb 21;15:04055. doi: 10.7189/jogh.15.04055 (PMC11842004; doi:10.7189/jogh.15.04055)
Supplement: Online Supplementary Document [file jogh-15-04055-s001.pdf]

Supplement to: Song Q, Lin L, Li T, Zhang P, Zeng Y, Deng D, Yi R, Liu D, Chen Y, Cai S, Chen P, Liu C. The treatment responses among different inhalation therapies for GOLD group E patients with chronic obstructive pulmonary disease. J Glob Health. 2025;15:04055.

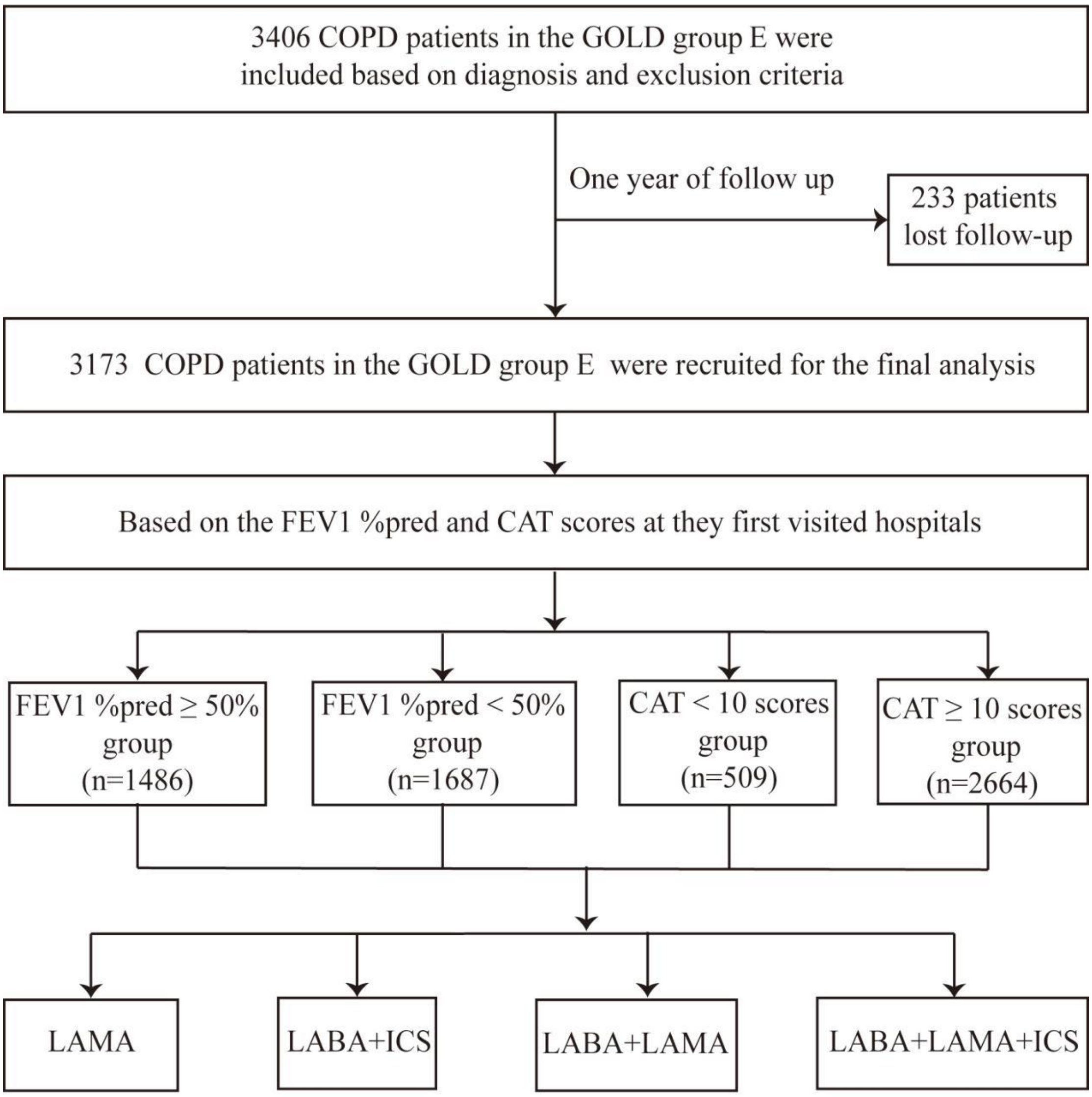

Figure S1. Flow chart of this study. COPD, Chronic Obstructive Pulmonary Disease; CAT, COPD Assessment Test; FEV1 %pred, Forced Expiratory Volume in the first second percentage of predicted; GOLD, Global Initiative for Chronic Obstructive Lung Disease; ICS, Inhaled Corticosteroid; LAMA, Long-Acting Muscarinic Antagonist; LABA, Long-Acting β2-Agonist.

Table S1. The results of collinearity test among the variables in the logistic regression model

Variables	Tolerance	VIF
Age	0.878	1.139
Sex	0.644	1.553
BMI	0.900	1.111
Smoke history	0.668	1.497
Biofuel exposure	0.932	1.073
FEV1 %pred	0.316	3.166
FEV1/FVC	0.324	3.082
CAT	0.645	1.550
mMRC	0.595	1.679
Exacerbations in the past year	0.966	1.036
Chronic heart disease	0.960	1.042
Hypertension	0.949	1.054
Diabetes	0.977	1.024

Abbreviations: BMI, Body Mass Index; CAT, COPD Assessment Test; FEV1 %pred, Forced Expiratory Volume in the first second percentage of predicted; FVC, Forced Vital Capacity; mMRC, modified Medical Research Council; VIF, Variance Inflation Factor.

Table S2. Multivariate analysis for the FEV1 %pred ≥ 50% group

Variables	Exacerbations		Frequent exacerbations		Hospitalizations		All cause of mortality	
	**aOR (95%CI)**	***aP*-value**	**aOR (95%CI)**	***aP*-value**	**aOR (95%CI)**	***aP*-value**	**aOR (95%CI)**	***aP*-value**
Therapy								
LABA+LAMA	Reference		Reference		Reference		Reference	
LAMA	1.28 (0.88-1.85)	0.198	1.24 (0.78-1.98)	0.356	0.94 (0.61-1.44)	0.773	0.60 (0.18-1.97)	0.401
LABA+ICS	1.08 (0.72-1.62)	0.710	1.07 (0.64-1.77)	0.805	0.76 (0.47-1.23)	0.262	1.03 (0.29-3.64)	0.965
LABA+LAMA+ICS	1.12 (0.78-1.61)	0.544	0.85 (0.53-1.35)	0.492	1.17 (0.77-1.77)	0.457	0.25 (0.06-1.01)	0.051
Age	1.00 (0.99-1.02)	0.747	1.00 (0.98-1.02)	0.988	1.01 (1.00-1.03)	0.105	1.08 (1.02-1.15)	**0.009**
Sex								
Male	Reference		Reference		Reference		Reference	
Female	0.80 (0.54-1.19)	0.272	0.68 (0.42-1.09)	0.111	0.82 (0.52-1.30)	0.405	1.09 (0.19-6.13)	0.922
BMI	1.01 (0.97-1.04)	0.742	0.99 (0.95-1.03)	0.655	0.99 (0.95-1.03)	0.642	0.93 (0.82-1.06)	0.285
Smoke history								
Never-smoker	Reference		Reference		Reference		Reference	
Former-smoker	0.96 (0.66-1.41)	0.848	0.83 (0.52-1.30)	0.411	0.86 (0.56-1.33)	0.508	1.46 (0.26-8.16)	0.665
Current-smoker	0.85 (0.59-1.23)	0.388	0.69 (0.44-1.07)	0.096	0.79 (0.52-1.20)	0.264	1.91 (0.38-9.55)	0.433
Biofuel exposure								
No	Reference		Reference		Reference		Reference	
Yes	1.04 (0.83-1.30)	0.731	1.02 (0.77-1.34)	0.911	1.00 (0.77-1.30)	0.995	0.91 (0.37-2.20)	0.827
FEV1 %pred	1.00 (0.99-1.01)	0.387	0.99 (0.98-1.01)	0.308	1.00 (0.99-1.01)	0.677	0.98 (0.95-1.02)	0.354
FEV1/FVC	0.99 (0.98-1.01)	0.483	1.01 (0.98-1.03)	0.609	0.99 (0.97-1.01)	0.343	1.09 (0.93-1.17)	0.074
CAT	0.99 (0.97-1.01)	0.483	1.01 (0.99-1.04)	0.284	0.98 (0.95-1.00)	0.054	1.08 (0.99-1.18)	0.067
mMRC	1.09 (0.95-1.25)	0.202	1.09 (0.92-1.29)	0.326	1.15 (0.98-1.34)	0.080	1.08 (0.63-1.86)	0.776
Exacerbations in the past year	1.03 (0.99-1.06)	0.132	1.06 (1.03-1.10)	**0.001**	0.98 (0.94-1.02)	0.362	1.08 (1.00-1.17)	0.054
Prescription outcomes								

Adjust treatment	Reference		Reference		Reference		Reference	
Continuous using	0.76 (0.60-0.97)	**0.026**	0.82 (0.61-1.11)	0.202	0.70 (0.53-0.92)	**0.010**	2.51 (0.72-8.78)	0.148
Comorbidities								
Chronic heart disease	1.62 (0.79-3.31)	0.185	0.77 (0.28-2.11)	0.617	1.75 (0.82-3.71)	0.147	2.05 (0.35-12.10)	0.429
Hypertension	0.72 (0.40-1.29)	0.273	1.16 ()0.57-2.35	0.678	0.82 (0.43-1.59)	0.564	0.87 (0.09-8.47)	0.904
Diabetes	0.61 (0.25-1.52)	0.291	0.41 (0.09-1.78)	0.233	1.11 (0.43-2.91)	0.828	0 (0-Inf)	0.991

Notes: Factors in the logistic model: therapy, prescription outcomes, age, sex, BMI, smoke history, biofuel exposure, FEV1%, FEV1/FVC, CAT, mMRC, exacerbations in the past year, and comorbidities. The bold P values indicate statistical significance.

Abbreviations: BMI, Body Mass Index; CI, Confidence Interval; COPD, Chronic Obstructive Pulmonary Disease; CAT, COPD Assessment Test; FEV1 %pred, Forced Expiratory Volume in the first second percentage of predicted; FVC, Forced Vital Capacity; ICS, Inhaled Corticosteroid; LAMA, Long-Acting Muscarinic Antagonist; LABA, Long-Acting β2-Agonist; mMRC, modified Medical Research Council; aOR, Adjusted odds Ratio.

Table S3. Multivariate analysis for the FEV1 %pred ≥ 50% group

**Variables**	**Exacerbations**		**Frequent exacerbations**		**Hospitalizations**		**All cause of mortality**	
	**aOR (95%CI)**	***aP*-value**	**aOR (95%CI)**	***aP*-value**	**aOR (95%CI)**	***aP*-value**	**aOR (95%CI)**	***aP*-value**
Therapy								
LABA+LAMA+ICS	Reference		Reference		Reference		Reference	
LABA+LAMA	0.89 (0.62-1.29)	0.544	1.18 (0.74-1.87)	0.492	0.85 (0.56-1.29)	0.457	4.00 (0.99-16.16)	0.051
LABA+ICS	0.96 (0.71-1.31)	0.819	1.25 (0.85-1.86)	0.256	0.64 (0.44-1.01)	0.053	4.12 (0.99-16.28)	0.054
LAMA	1.14 (0.87-1.49)	0.340	1.46 (0.99-1.86)	0.056	0.80 (0.59-1.09)	0.165	2.41 (0.67-8.71)	0.181

Notes: Factors in the logistic model: therapy, prescription outcomes, age, sex, BMI, smoke history, biofuel exposure, FEV1%, FEV1/FVC, CAT, mMRC, exacerbations in the past year, and comorbidities.

Abbreviations: BMI, Body Mass Index; CI, Confidence Interval; COPD, Chronic Obstructive Pulmonary Disease; CAT, COPD Assessment Test; FEV1 %pred, Forced Expiratory Volume in the first second percentage of predicted; FVC, Forced Vital Capacity; ICS, Inhaled Corticosteroid; LAMA, Long-Acting Muscarinic Antagonist; LABA, Long-Acting β2-Agonist; mMRC, modified Medical Research Council; aOR, Adjusted odds Ratio.

Table S4. Multivariate analysis for the FEV1 %pred < 50% group

Variables	Exacerbations		Frequent exacerbations		Hospitalizations		All cause of mortality	
	**aOR (95%CI)**	***aP*-value**	**aOR (95%CI)**	***aP*-value**	**aOR (95%CI)**	***aP*-value**	**aOR (95%CI)**	***aP*-value**
Therapy								
LABA+LAMA	Reference		Reference		Reference		Reference	
LAMA	1.74 (1.17-2.61)	**0.007**	2.55 (1.53-4.26)	**<0.001**	1.16 (0.73-1.87)	0.530	0.92 (0.27-3.16)	0.897
LABA+ICS	1.48 (1.01-2.35)	**0.015**	2.27 (1.27-4.06)	**0.006**	1.31 (0.76-2.25)	0.335	0.40 (0.07-2.31)	0.304
LABA+LAMA+ICS	1.00 (0.72-1.41)	0.980	1.40 (0.89-2.22)	0.145	1.19 (0.80-1.76)	0.393	0.84 (0.31-2.27)	0.730
Age	0.99 (0.98-1.01)	0.341	1.00 (0.98-1.01)	0.535	1.02 (1.00-1.03)	**0.046**	1.10 (1.05-1.15)	**<0.001**
Sex								
Male	Reference		Reference		Reference		Reference	
Female	0.98 (0.64-1.52)	0.941	0.68 (0.40-1.18)	0.175	1.13 (0.68-1.86)	0.646	0.16 (0.02-1.39)	0.096
BMI	0.98 (0.95-1.01)	0.191	0.99 (0.95-1.02)	0.491	0.99 (0.95-1.02)	0.486	1.05 (0.97-1.14)	0.256
Smoke history								
Never-smoker	Reference		Reference		Reference		Reference	
Former-smoker	1.24 (0.87-1.77)	0.233	1.08 (0.71-1.65)	0.710	1.15 (0.76-1.74)	0.512	0.73 (0.26-2.04)	0.543
Current-smoker	1.15 (0.80-1.64)	0.453	0.96 (0.63-1.47)	0.856	1.14 (0.75-1.73)	0.531	0.96 (0.33-2.78)	0.938
Biofuel exposure								
No	Reference		Reference		Reference		Reference	
Yes	0.77 (0.62-1.12)	0.057	0.83 (0.64-1.08)	0.163	0.87 (0.68-1.11)	0.247	1.87 (1.01-3.46)	**0.046**
FEV1 %pred	0.99 (0.98-1.01)	0.364	0.98 (0.97-1.00)	0.048	1.00 (0.98-1.02)	0.974	0.93 (0.89-0.97)	**0.001**
FEV1/FVC	1.01 (0.99-1.02)	0.353	1.01 (0.99-1.03)	0.291	0.99 (0.97-1.01)	0.364	1.02 (0.97-1.06)	0.426
CAT	1.00 (0.98-1.02)	0.835	1.01 (0.99-1.03)	0.471	1.00 (0.98-1.03)	0.708	1.05 (0.99-1.11)	0.091
mMRC	1.10 (0.97-1.25)	0.155	1.09 (0.94-1.28)	0.259	1.21 (1.04-1.40)	**0.014**	1.15 (0.76-1.74)	0.506
Exacerbations in the past year	1.00 (0.97-1.03)	0.984	1.03 (0.99-1.06)	0.101	0.96 (0.93-1.00)	0.057	1.04 (0.98-1.11)	0.174
Prescription outcomes								

Adjust treatment	Reference		Reference		Reference		Reference	
Continuous using	0.53 (0.42-0.68)	**<0.001**	0.67 (0.51-0.90)	**0.007**	0.54 (0.41-0.71)	**<0.001**	0.89 (0.42-1.90)	0.765
Comorbidities								
Chronic heart disease	0.99 (0.54-1.84)	0.987	0.81 (0.37-1.78)	0.598	0.81 (0.39-1.68)	0.570	3.81 (1.29-11.22)	**0.015**
Hypertension	1.66 (0.87-3.17)	0.126	1.52 (0.72-3.24)	0.273	1.55 (0.76-3.15)	0.230	1.37 (0.28-6.60)	0.698
Diabetes	1.39 (0.49-3.94)	0.540	2.75 (0.94-8.03)	0.065	0.87 (0.24-3.20)	0.836	2.60 (0.29-23.24)	0.392

Notes: Factors in the logistic model: therapy, prescription outcomes, age, sex, BMI, smoke history, biofuel exposure, FEV1%, FEV1/FVC, CAT, mMRC, exacerbations in the past year, and comorbidities. The bold P values indicate statistical significance.

Abbreviations: BMI, Body Mass Index; CI, Confidence Interval; COPD, Chronic Obstructive Pulmonary Disease; CAT, COPD Assessment Test; FEV1 %pred, Forced Expiratory Volume in the first second percentage of predicted; FVC, Forced Vital Capacity; ICS, Inhaled Corticosteroid; LAMA, Long-Acting Muscarinic Antagonist; LABA, Long-Acting β2-Agonist; mMRC, modified Medical Research Council; aOR, Adjusted odds Ratio.

Table S5. Multivariate analysis for the FEV1 %pred < 50% group

Variables	Exacerbations		Frequent exacerbations		Hospitalizations		All cause of mortality	
	**aOR (95%CI)**	***aP*-value**	**aOR (95%CI)**	***aP*-value**	**aOR (95%CI)**	***aP*-value**	**aOR (95%CI)**	***aP*-value**
Therapy								
LABA+LAMA+ICS	Reference		Reference		Reference		Reference	
LABA+LAMA	1.00 (0.71-1.39)	0.980	0.71 (0.45-1.12)	0.145	0.84 (0.57-1.25)	0.393	1.19 (0.44-3.22)	0.730
LABA+ICS	1.47 (1.01-2.13)	**0.043**	1.62 (1.06-2.47)	**0.027**	1.10 (0.72-1.69)	0.659	0.47 (0.10-2.18)	0.338
LAMA	1.74 (1.31-2.31)	**<0.001**	1.81 (1.32-2.50)	**<0.001**	0.98 (0.70-1.37)	0.908	1.10 (0.46-2.65)	0.836

Notes: Factors in the logistic model: therapy, prescription outcomes, age, sex, BMI, smoke history, biofuel exposure, FEV1%, FEV1/FVC, CAT, mMRC, exacerbations in the past year, and comorbidities. The bold P values indicate statistical significance.

Abbreviations: BMI, Body Mass Index; CI, Confidence Interval; COPD, Chronic Obstructive Pulmonary Disease; CAT, COPD Assessment Test; FEV1 %pred, Forced Expiratory Volume in the first second percentage of predicted; FVC, Forced Vital Capacity; ICS, Inhaled Corticosteroid; LAMA, Long-Acting Muscarinic Antagonist; LABA, Long-Acting β2-Agonist; mMRC, modified Medical Research Council; aOR, Adjusted odds Ratio.

Table S6. Multivariate analysis for the CAT < 10 scores group

**Variables**	**Exacerbations**		**Frequent exacerbations**		**Hospitalizations**		**All cause of mortality**	
	**aOR (95%CI)**	***aP*-value**	**aOR (95%CI)**	***aP*-value**	**aOR (95%CI)**	***aP*-value**	**aOR (95%CI)**	***aP*-value**
Therapy								
LABA+LAMA	Reference		Reference		Reference		N/A	
LAMA	0.76 (0.41-1.42)	0.388	0.90 (0.40-2.02)	0.799	0.83 (0.41-1.69)	0.606	N/A	N/A
LABA+ICS	0.62 (0.32-1.18)	0.147	0.96 (0.42-2.19)	0.920	0.59 (0.28-1.27)	0.179	N/A	N/A
LABA+LAMA+ICS	0.89 (0.49-1.62)	0.706	0.76 (0.34-1.66)	0.486	1.12 (0.57-2.18)	0.750	N/A	N/A
Age	1.01 (0.99-1.03)	0.466	1.01 (0.98-1.04)	0.487	1.03 (1.00-1.06)	0.019	N/A	N/A
Sex								
Male	Reference		Reference		Reference		N/A	
Female	1.35 (0.64-2.87)	0.429	1.14 (0.42-3.11)	0.798	1.11 (0.48-2.61)	0.802	N/A	N/A
BMI	1.01 (0.96-1.08)	0.632	1.02 (0.95-1.10)	0.585	1.00 (0.93-1.07)	0.998	N/A	N/A
Smoke history								
Never-smoker	Reference		Reference	6	Reference		N/A	
Former-smoker	0.81 (0.42-1.56)	0.527	0.95 (0.40-2.26)	0.900	0.78 (0.38-1.63)	0.510	N/A	N/A
Current-smoker	0.99 (0.53-1.86)	0.980	1.11 (0.48-2.56)	0.812	0.82 (0.40-1.67)	0.580	N/A	N/A
Biofuel exposure								
No	Reference		Reference		Reference		N/A	
Yes	1.13 (0.76-1.68)	0.548	1.03 (0.61-1.74)	0.915	1.09 (0.70-1.72)	0.700	N/A	N/A
FEV1 %pred	1.00 (0.99-1.02)	0.706	0.99 (0.97-1.01)	0.270	1.00 (0.98-1.02)	0.925	N/A	N/A
FEV1/FVC	1.01 (0.98-1.03)	0.694	1.01 (0.98-1.05)	0.537	1.00 (0.97-1.04)	0.817	N/A	N/A
CAT	1.08 (0.98-1.18)	0.115	1.15 (1.01-1.32)	**0.035**	1.05 (0.94-1.17)	0.378	N/A	N/A
mMRC	1.33 (1.03-1.71)	**0.026**	1.27 (0.91-1.76)	0.158	1.17 (0.88-1.56)	0.276	N/A	N/A
Exacerbations in the past year	1.09 (0.99-1.19)	0.065	1.09 (0.99-1.20)	0.075	0.95 (0.86-1.06)	0.354	N/A	N/A
Prescription outcomes								

Adjust treatment	Reference		Reference		Reference		N/A	
Continuous using	0.72 (0.47-1.10)	0.124	0.94 (0.54-1.64)	0.837	0.79 (0.49-1.27)	0.329	N/A	N/A
Comorbidities								
Chronic heart disease	1.03 (0.17-6.22)	0.976	1.54 (0.16-14.54)	0.706	0.48 (0.05-4.60)	0.528		
Hypertension	0.37 (0.07-1.87)	0.229	0.52 (0.06-4.38)	0.551	0.67 (0.14-3.26)	0.616		
Diabetes	0.65 (0.17-2.59)	0.544	1.27 (0.26-6.27)	0.773	1.34 (0.33-5.35)	0.682		

Notes: Factors in the logistic model: therapy, prescription outcomes, age, sex, BMI, smoke history, biofuel exposure, FEV1%, FEV1/FVC, CAT, mMRC, exacerbations in the past year, and comorbidities. N/A, Not applicable. The bold P values indicate statistical significance.

Abbreviations: BMI, Body Mass Index; CI, Confidence Interval; COPD, Chronic Obstructive Pulmonary Disease; CAT, COPD Assessment Test; FEV1 %pred, Forced Expiratory Volume in the first second percentage of predicted; FVC, Forced Vital Capacity; ICS, Inhaled Corticosteroid; LAMA, Long-Acting Muscarinic Antagonist; LABA, Long-Acting β2-Agonist; mMRC, modified Medical Research Council; aOR, Adjusted odds Ratio.

Table Ss7. Multivariate analysis for the CAT < 10 scores group

**Variables**	**Exacerbations**		**Frequent exacerbations**		**Hospitalizations**		**All cause of mortality**	
	**aOR (95%CI)**	***aP*-value**	**aOR (95%CI)**	***aP*-value**	**aOR (95%CI)**	***aP*-value**	**aOR (95%CI)**	***aP*-value**
Therapy								
LABA+LAMA+ICS	Reference		Reference		Reference		N/A	
LABA+LAMA	1.12 (0.62-2.04)	0.706	1.32 (0.60-2.90)	0.486	0.90 (0.46-1.75)	0.750	N/A	N/A
LABA+ICS	0.69 (0.41-1.19)	0.184	1.27 (0.63-2.53)	0.503	0.53 (0.28-1.01)	0.053	N/A	N/A
LAMA	0.85 (0.51-1.43)	0.546	1.19 (0.60-2.36)	0.618	0.74 (0.41-1.34)	0.323	N/A	N/A

Notes: Factors in the logistic model: therapy, prescription outcomes, age, sex, BMI, smoke history, biofuel exposure, FEV1%, FEV1/FVC, CAT, mMRC, exacerbations in the past year, and comorbidities. N/A, Not applicable.

Abbreviations: BMI, Body Mass Index; CI, Confidence Interval; COPD, Chronic Obstructive Pulmonary Disease; CAT, COPD Assessment Test; FEV1 %pred, Forced Expiratory Volume in the first second percentage of predicted; FVC, Forced Vital Capacity; ICS, Inhaled Corticosteroid; LAMA, Long-Acting Muscarinic Antagonist; LABA, Long-Acting β2-Agonist; mMRC, modified Medical Research Council; aOR, Adjusted odds Ratio.

Table S8. Multivariate analysis for the CAT ≥ 10 scores group

Variables	Exacerbations		Frequent exacerbations		Hospitalizations		All cause of mortality	
	**aOR (95%CI)**	***aP*-value**	**aOR (95%CI)**	***aP*-value**	**aOR (95%CI)**	***aP*-value**	**aOR (95%CI)**	***aP*-value**
Therapy								
LABA+LAMA	Reference		Reference		Reference		Reference	
LAMA	1.69 (1.24-2.29)	**0.001**	2.01 (1.37-2.95)	**<0.001**	1.08 (0.76-1.55)	0.658	0.74 (0.31-1.78)	0.502
LABA+ICS	1.48 (1.05-2.09)	**0.024**	1.73 (1.12-2.66)	**0.013**	1.09 (0.73-1.63)	0.680	0.72 (0.25-2.09)	0.551
LABA+LAMA+ICS	1.09 (0.83-1.43)	0.526	1.18 (0.83-1.69)	0.355	1.20 (0.88-1.64)	0.259	0.61 (0.29-1.28)	0.191
Age	1.00 (0.99-1.01)	0.571	0.99 (0.98-1.01)	0.359	1.01 (1.00-1.02)	**0.049**	1.08 (1.04-1.11)	**<0.001**
Sex								
Male	Reference		Reference		Reference		Reference	
Female	0.86 (0.63-1.18)	0.354	0.67 (0.46-0.98)	**0.038**	0.95 (0.66-1.37)	0.786	0.47 (0.14-1.52)	0.205
BMI	0.99 (0.97-1.01)	0.422	0.99 (0.96-1.01)	0.345	0.99 (0.96-1.02)	0.437	1.01 (0.94-1.08)	0.863
Smoke history								
Never-smoker	Reference		Reference		Reference		Reference	
Former-smoker	1.15 (0.87-1.52)	0.329	0.95 (0.68-1.31)	0.749	1.07 (0.77-1.48)	0.695	1.00 (0.40-2.50)	0.995
Current-smoker	0.99 (0.75-1.31)	0.942	0.79 (0.57-1.09)	0.145	1.01 (0.73-1.40)	0.930	1.33 (0.53-3.34)	0.545
Biofuel exposure								
No	Reference		Reference		Reference		Reference	
Yes	0.87 (0.73-1.03)	0.099	0.91 (0.75-1.12)	0.383	0.92 (0.76-1.11)	0.390	1.52 (0.91-2.53)	0.109
FEV1 %pred	0.99 (0.99-1.00)	0.163	0.99 (0.98-1.00)	0.093	1.00 (0.99-1.01)	0.765	0.97 (0.94-0.99)	**0.007**
FEV1/FVC	1.00 (0.99-1.01)	0.667	1.01 (0.99-1.02)	0.337	0.99 (0.98-1.01)	0.318	1.03 (1.00-1.07)	0.065
CAT	1.00 (0.98-1.02)	0.816	1.01 (0.99-1.03)	0.387	0.99 (0.98-1.01)	0.584	1.06 (1.01-1.11)	**0.020**
mMRC	1.05 (0.95-1.16)	0.317	1.07 (0.95-1.21)	0.262	1.18 (1.05-1.32)	**0.006**	1.16 (0.83-1.60)	0.383
Exacerbations in the past year	1.00 (0.98-1.02)	0.840	1.04 (1.01-1.06)	**0.005**	0.97 (0.94-1.00)	0.052	1.06 (1.01-1.11)	**0.010**
Prescription outcomes								

Adjust treatment	Reference		Reference		Reference		Reference	
Continuous using	0.63 (0.52-0.76)	**<0.001**	0.72 (0.57-0.89)	**0.003**	0.59 (0.48-0.73)	**<0.001**	1.22 (0.63-2.34)	0.552
Comorbidities								
Chronic heart disease	1.23 (0.76-1.99)	0.394	0.76 (0.40-1.45)	0.405	1.23 (0.72-2.08)	0.448	3.18 (1.31-7.69)	**0.010**
Hypertension	1.18 (0.75-1.85)	0.474	1.44 (0.85-2.45)	0.179	1.16 (0.70-1.92)	0.567	1.04 (0.29-3.64)	0.957
Diabetes	1.02 (0.47-2.22)	0.963	1.13 (0.44-2.85)	0.801	0.96 (0.38-2.45)	0.940	1.43 (0.18-11.61)	0.740

Notes: Factors in the logistic model: therapy, prescription outcomes, age, sex, BMI, smoke history, biofuel exposure, FEV1%, FEV1/FVC, CAT, mMRC, exacerbations in the past year, and comorbidities. The bold P values indicate statistical significance.

Abbreviations: BMI, Body Mass Index; CI, Confidence Interval; COPD, Chronic Obstructive Pulmonary Disease; CAT, COPD Assessment Test; FEV1 %pred, Forced Expiratory Volume in the first second percentage of predicted; FVC, Forced Vital Capacity; ICS, Inhaled Corticosteroid; LAMA, Long-Acting Muscarinic Antagonist; LABA, Long-Acting β2-Agonist; mMRC, modified Medical Research Council; aOR, Adjusted odds Ratio.

Table S9. Multivariate analysis for th CAT ≥ 10 scores group

**Variables**	**Exacerbations**		**Frequent exacerbations**		**Hospitalizations**		**All cause of mortality**	
	**aOR (95%CI)**	***aP*-value**	**aOR (95%CI)**	***aP*-value**	**aOR (95%CI)**	***aP*-value**	**aOR (95%CI)**	***aP*-value**
Therapy								
LABA+LAMA+ICS	Reference		Reference		Reference		Reference	
LABA+LAMA	0.92 (0.70-1.20)	0.526	0.85 (0.59-1.21)	0.355	0.83 (0.61-1.14)	0.259	1.64 (0.78-3.45)	0.191
LABA+ICS	1.36 (1.04-1.77)	**0.023**	1.46 (1.07-2.00)	**0.018**	0.91 (0.66-1.24)	0.549	1.19 (0.48-2.95)	0.709
LAMA	1.51 (1.23-1.86)	**<0.001**	1.70 (1.33-2.17)	**<0.001**	0.90 (0.71-1.15)	0.418	1.21 (0.61-2.40)	0.577

Notes: Factors in the logistic model: therapy, prescription outcomes, age, sex, BMI, smoke history, biofuel exposure, FEV1%, FEV1/FVC, CAT, mMRC, exacerbations in the past year, and comorbidities. The bold P values indicate statistical significance.

Abbreviations: BMI, Body Mass Index; CI, Confidence Interval; COPD, Chronic Obstructive Pulmonary Disease; CAT, COPD Assessment Test; FEV1 %pred, Forced Expiratory Volume in the first second percentage of predicted; FVC, Forced Vital Capacity; ICS, Inhaled Corticosteroid; LAMA, Long-Acting Muscarinic Antagonist; LABA, Long-Acting β2-Agonist; mMRC, modified Medical Research Council; aOR, Adjusted odds Ratio.
